# Impact of Spray Concentration and Application Frequency to Modulate Phosphonic Acid Residues in Container-Grown Grapevines

**DOI:** 10.1021/acs.jafc.4c12228

**Published:** 2025-04-18

**Authors:** Sören Otto, Beate Berkelmann-Löhnertz, Bianca May, Randolf Kauer, Ralf Schweiggert

**Affiliations:** † Department of Beverage Research, Chair of Analysis & Technology of Plant-based Foods Geisenheim University, Von-Lade-Street 1, Geisenheim D-65366, Germany; ‡ Department of Crop Protection, Chair of Crop Protection in Viticulture and Horticulture Geisenheim University, Von-Lade-Street 1, Geisenheim D-65366, Germany; § Department of Enology, Chair of Wine and Beverage Chemistry Geisenheim University, Von-Lade-Street 1, Geisenheim D-65366, Germany; ∥ Department of Viticulture, Chair of Organic Viticulture Geisenheim University, Von-Lade-Street 1, Geisenheim D-65366, Germany

**Keywords:** organic horticulture, pesticide residues, food safety, crop protection, *Vitis vinifera* L.

## Abstract

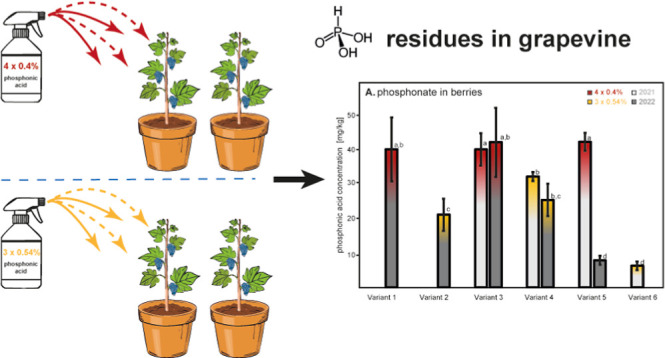

This study investigated the translocation and persistence of inorganic phosphonate in container-grown vines of *Vitis vinifera* L. cv. Riesling after foliar and soil applications over two consecutive years. Phosphonate concentrations were monitored in leaves, petioles, grape canes, shoot tips, inflorescences, and berries during the season, applying an identical total amount of 3 or 4 sprays of 0.54 or 0.4% (w/v, aq) phosphonate, respectively. The overall uptake of inorganic phosphonate into the leaves was either identical (year 1) or substantially lower (year 2) when spraying 3 times (0.54%) instead of 4 times (0.4%) as expressed by the area under the concentration vs time curve. Residues found in leaves at the end of the vegetation period were also lower when spraying 3 times. Across both years, residues in berries were also significantly lower when applying the 0.54% phosphonate solution (20.2–30.9 mg/kg) 3 times as compared with the 4× application of 0.4% phosphonate (38.5–40.6 mg/kg). Soil applications resulted in a comparably low overall uptake but still yielding measurable residues in berries (6.0 ± 1.2 mg/kg). Further data on grape cane, shoot tips, and inflorescences supported the hypothesis that phosphonate residues in the plant and, ultimately, in the berries and the resulting products might be significantly reduced when spraying 3 times (0.54%) instead of 4 times (0.4%).

## 1 Introduction

Phosphonic acid and its salts, particularly potassium hydrogen phosphonate (KH_2_PO_3_) and potassium phosphonate (K_2_HPO_3_), are widely used in horticulture. The compounds do not only boost plant vigor but also have a fungistatic effect, mainly by activating the plant defense system.^1,2^ For instance, they have been shown to support controlling pathogens such as *Phytophthora infestans* on potatoes and *Plasmopara viticola* on grapevines.^3−5^ However, the distribution and eventual accumulation of inorganic phosphonate within the plant system and, particularly, in perennial crops like grapevines are yet poorly understood.^6^ A better understanding therein might support ensuring high efficacy while minimizing residues of phosphonic acid in agricultural products.

Phosphonates, employed across more than 100 crops worldwide, are critical in pest management strategies due to their effectiveness and safety profile, including minimal residual toxicity. Nevertheless, worldwide institutions like FAO, USDA, USEPA, and the EU have set ambitious goals to enhance agricultural sustainability and, in particular, to reduce chemical residues in food chains and the environment.^2,7^ Inorganic phosphonate played a particular role in organic farming in the EU, where it had been applied alongside copper-based fungicides to assist plant defense against several pathogens like downy mildew on grapevines until 2013. Well-quantifiable residues of phosphonate in grapevines and the resulting wines, its synthetic origin, and its direct effect on pathogenic fungi urged the European Union to ban phosphonate usage from organic farming (Commission Implementing Regulation (EU) No. 369/2013).^7,8^ Despite the ban, phosphonate residues were eventually found in organic products, but it is subject of ongoing discussions whether such residues might solely be the consequence of foliar applications or might also result from persistent residues in the soil, groundwater, or perennial wooden parts of the plant.^9,10^ Additionally, organic and mineral-based fertilizers may contain trace amounts of phosphonic acid, while microbial phosphate reduction might further contribute to measurable residues in plants.^11^ A recent study also identified diammonium phosphate, a common nitrogen-rich fermentation aid for yeast, as a potential source of significant phosphonate impurities.^12^ Recent advances have enabled the differentiation of foliar application residues from other sources.^13^ Noteworthy, even trace residues may result in noncompliance with organic certification standards and lead to economic losses.^11^

Therefore, the challenge of minimizing the use of pesticides like inorganic phosphonate in agriculture is a worldwide concern, particularly in regions with humid climates where fungal pressure is high. An improved understanding of the persistence of the pesticides and improved strategies for their application are most urgently required. For these purposes, our study focused on unraveling the distribution of inorganic phosphonate in grapevines (*Vitis vinifera* L.) after conducting various variants of foliar applications. The study was also complemented by soil uptake experiments. In particular, new application regimes such as spraying more concentrated solutions at lesser frequencies were investigated by monitoring phosphonate distribution and, ultimately, its transfer to the berries. For this purpose, inorganic phosphonate was applied to vines potted in small containers by implementing 4 or 3 treatments with two different phosphonic acid concentrations (0.40% and 0.54%, w/w), respectively. All applications were carried out before the phenological stage of flowering. Applying the more concentrated solution with 3 treatments allowed to have the last treatment 14 days before full bloom, being only 2 days before full bloom when applying 4 treatments. In addition, some variants were sprayed for two consecutive years to study the accumulation of phosphonate within the vine, while others were only sprayed in the first but not in the second year to study phosphonate elimination from the plant. Samples of various plant parts including leaves, petioles, berries, grape canes, shoot tips, and inflorescences were taken throughout the full growth and ripening period of the two studied consecutive years, then being analyzed for inorganic phosphonate by IC-ICP-MS.

## 2 Materials and Methods

### 2.1 Grapevine Cultivation

One year-old grapevine shoots of *V. vinifera* L. cv. Riesling were sourced from an untainted trial and were grafted onto *Vitis riparia* × *Vitis cinerea* cv. Börner rootstocks by the Department of Grapevine Breeding at Geisenheim University. In spring 2020, these grafted shoots were then planted in containers measuring 28 × 22.5 × 28 cm^3^ with a standard soil blend (ED73, Einheitserde, Sinntal, Germany), without detectable phosphonate residues, and treated according to common commercial practice without any use of products containing phosphonic acid. The grapevines were supported by a trellis system without a fruiting wire. In total, 96 grapevines were planted, including two buffer vines for separation of individual treatments. From the 2021 growing season onward, the vines were irrigated daily with a single-drop system delivering 2 L of water per pot. They were also supplemented in mid-May with 25 g per pot of Basacote Plus 6 M (Compo Expert, Münster, Germany), a long-term stable fertilizer that provided essential nutrients (nitrogen, phosphorus, and potassium), and micronutrients (B, Cu, Fe, Mn, Mo, and Zn). Crop protection management included two fungicide treatments (Sercadis, BASF SE, Ludwigshafen, Germany) to control powdery mildew and two insecticide treatments (Confidor, Bayer CropScience, Leverkusen, Germany) from growth stages 19 to 36 (E-L stages, as defined by Coombe^14^). This specific plant protection was conducted according to weather conditions and predicted infections provided by the decision support systems of Geisenheim University.^15^ Meteorological data concerning temperature, precipitation, and sunshine hours were recorded by a weather station of the German Meteorological Service located near the experimental site in Geisenheim.^16^ The trial has been described by us earlier, and part of the data was utilized for the development of an index demonstrating the origin of phosphonate found in *V. vinifera* leaves and petioles.^13^

### 2.2 Phosphonic Acid Applications

**Figure 1:**
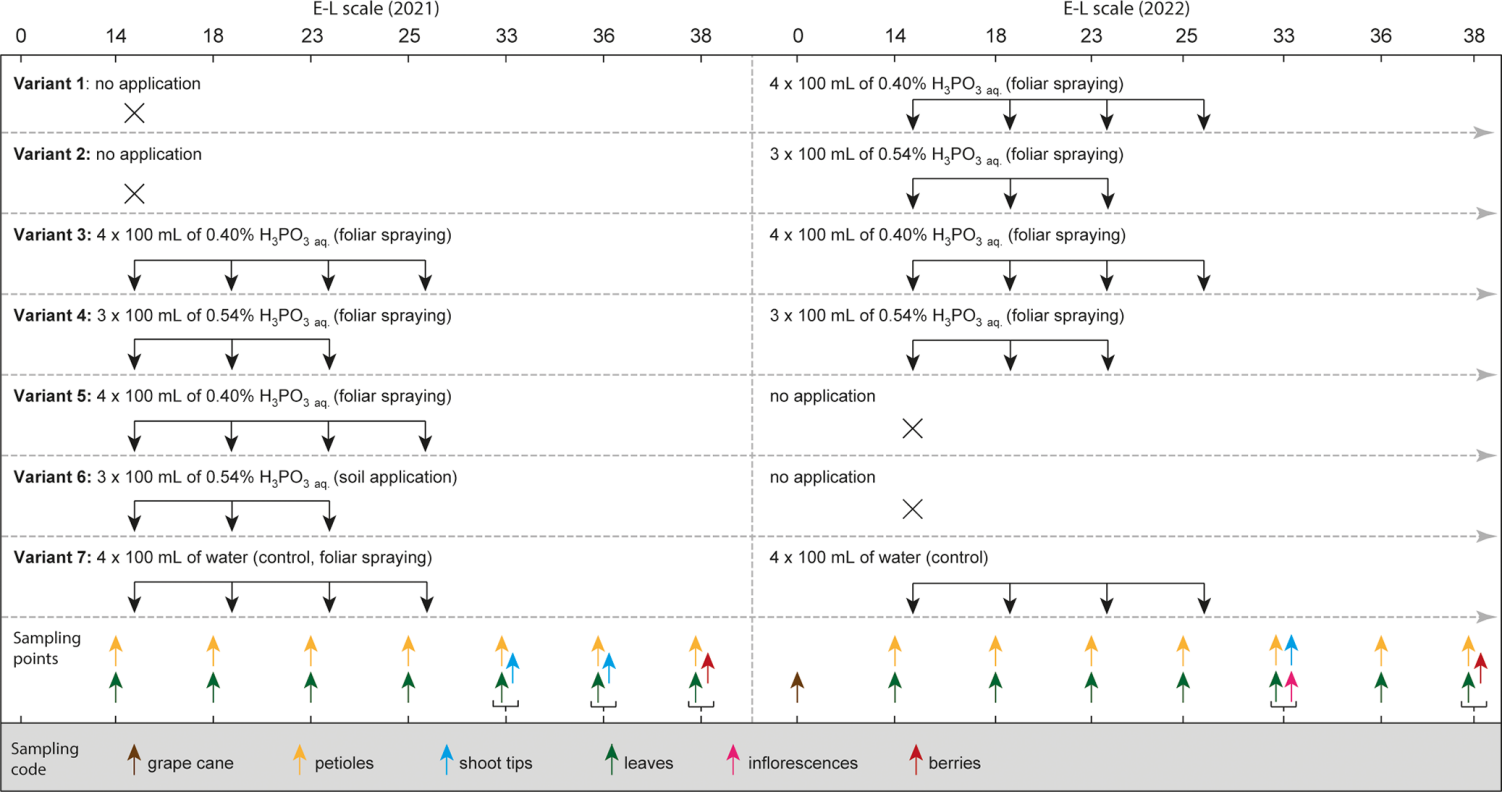
Experimental design of the trial. Arrows down indicate at which E-L stadium the spray treatments were applied. The applications were performed shortly after the sampling in E-L 14, 18, 23, and 25 in each year. Arrows up indicate at which E-L stadium samples were taken. All variants were sampled according to the planned scheme, except for Variant 6, which was only sampled in the first year. Besides the shown sampling in February 2022, grape cane was also sampled in February 2023 (not shown).

The experimental design involving seven different phosphonate treatment variants is summarized in Figure 1. Variants 1, 2, and 7 were not sprayed with phosphonate at all in the first year (2021). In the second year (2022), Variant 1 was sprayed with a 0.4% (w/v) phosphonic acid solution at 4 time points, while Variant 2 received a 0.54% solution at 3 time points. The results of these two variants of 2022 represented repetitions of the results derived from variants 3 and 4 in 2021 (see below). Variants 3 and 4 were sprayed with 0.4 and 0.54% for 4 and 3 times in both years to study phosphonate accumulation over two years. Variant 5 received the 0.4% phosphonate solution for 4 times only in 2021 but not in 2022 to study phosphonate depletion. Variant 6 received a 0.54% phosphonate solution applied only in 2021 and directly to the soil to study another uptake route. Variant 7 was a control treatment without any applications during both years (4 times control treatments with water and without any phosphonate application).

For the application of phosphonate, phosphonic acid (99%, w/w, Sigma-Aldrich, Steinheim, Germany) was diluted with deionized water to 0.4 or 0.54% (w/v) prior to spraying. Each application involved spraying approximately 100 mL of this solution onto a container vine using a Mesto 3610 high-pressure sprayer (Mesto, Freiburg, Germany), a process completed in roughly 13 s at an initial pressure of 6 bar, comparable to field applications. The 0.4% solution of phosphonic acid was administered 4 times as described above, with each application spaced 2 weeks apart, consequently E-L stages 15, 18, 23, and 26. In contrast, the higher concentration of 0.54% was sprayed only 3 times at the same two week intervals, also beginning at E-L 15. Spraying treatments as described were stopped after the mid of full flowering (E-L 23, 3 × 0.54%) and the end of full flowering (E-L 26, 4 × 0.4%). In order to ensure that phosphonic acid was available solely through root uptake, 100 mL of the 0.54% solution was evenly distributed carefully over the soil surface of the pot. Each treatment variant included 3 biological replicates each comprising 4 neighboring grapevine plants, totaling to 12 grapevines per variant. As shown in Figure 1, the trial was designed for two consecutive years.

### 2.3 Sample Collection

Systematic sampling was conducted by carefully selecting eight leaves along with their petioles from each directional side of a vine row, specifically from the north and south exposures. This should ensure a representative collection across the spatial orientation of the vines, yielding a total of 16 leaves with petioles gathered from each one of three biological replicates, each comprising a set of four vines to form a single pooled sample. Sampling was executed at predefined growth stages, namely, E-L 14, 18, 23, 25, 33, 36, and 38, i.e., over the entire growing season. For example, in 2021, a total of 21 leaf and 21 petiole samples were collected for each of the specified growth stages, resulting in 294 samples. In 2022, each of these growth stages yielded 18 leaf and 18 petiole samples (excluding Variant 6), totaling 252 samples, respectively. No soil treatment was applied in 2022. Consequently, no samples were collected from this treatment variant during this period. Postcollection, the samples were separated into leaves and petioles, frozen at −20 °C, and then freeze-dried (Beta 2–8 LD plus, Martin Christ, Osterode am Harz, Germany). For each replicate, eight inflorescences were collected in the E-L 23 stage in 2022. A 50 mL tube was placed over each inflorescence and gently shaken to completely detach the inflorescence. Contaminants such as petioles and insects were removed from the collected material manually, which was subsequently freeze-dried. Additionally, eight shoot tips per replicate were sampled at E-L 33 in both years and at E-L 36 only in 2021, by cutting 5–15 cm from each tip and processed similarly to the leaves and petioles. The dried samples were ground to a particle size of ≤0.5 mm using a laboratory mill (CT 293 Cyclotec, Foss, Hamburg, Germany). Grape cane samples were obtained by cutting 10 cm-long wooden shoots from one year-old shoots in February 2022 and 2023, and stored in cellulose bags. Grape canes were dried in a laboratory oven at 40 °C. The dried branches were then premilled using a cutting mill (SM 2000, Retsch, Haan, Germany) and subsequently ground to a particle size of ≤1.0 mm with the mentioned laboratory mill (Cyclotec). Berry sampling was carried out at the E-L 38 stage, where 80 berries were harvested from each of the three biological replicates (four vines). The berries were processed according to the method described by Otto et al.,^17^ all dried and ground materials were stored at room temperature until phosphonate analyses using IC-ICP-MS.

### 2.4 Analyses of Phosphonic Acid by IC-ICP-MS

In brief, 50 ± 0.5 mg of the freeze-dried sample was combined with 10 mL of ultrapure water and stirred for 4 h. When less sample material was available for inflorescence, sample amounts of 10–50 mg were used. After centrifuging for phase separation, a filtration of the supernatant through a 0.45 μm membrane filter was carried out (regenerated cellulose, GE Healthcare, Chicago, Illinois). After eventual dilution with water to achieve signals within the linear quantitation range, samples were subjected to our IC-ICP-MS system equipped with a Metrosep A Supp 10 column (4.6 μm, 100 mm × 4.0 mm i.d., Metrohm, Herisau, Switzerland) for quantitation of phosphonic acid using the ICP–MS detector.^17^

To ensure measurement accuracy, the detection limits for phosphonic acid in different tissues were determined. The limits of detection for leaf, petiole, and berries were 0.04, 0.03, and 0.05 mg/kg, and the limits of quantification were 0.12, 0.08, and 0.15 mg/kg, respectively.^17^ Results were expressed as mg phosphonic acid per kg of fresh matter (mg/kg) unless stated otherwise.

### 2.5 Statistical Analyses

Data was presented as mean values of phosphonic acid content in milligram per kilogram of fresh weight (mg/kg) with standard deviation unless stated otherwise. For this purpose, the dry matter content was determined gravimetrically in the course of freeze-drying. Statistical analyses were conducted, including analyses of variance (ANOVA) and posthoc Tukey tests for identifying statistically significant differences of means (*p* < 0.05), using Microsoft Excel (Microsoft, Redmond, Washington, USA). The area under the concentration vs time curve (AUC) as a further measure to estimate the overall phosphonate uptake or availability in a plant organ was determined by trapezoidal approximation and expressed in mg/kg × weeks (mg × wks/kg).

## 3 Results and Discussion

### 3.1 Effect of Treatment and Previous Year on Residues in Leaf and Petiole

**Figure 2:**
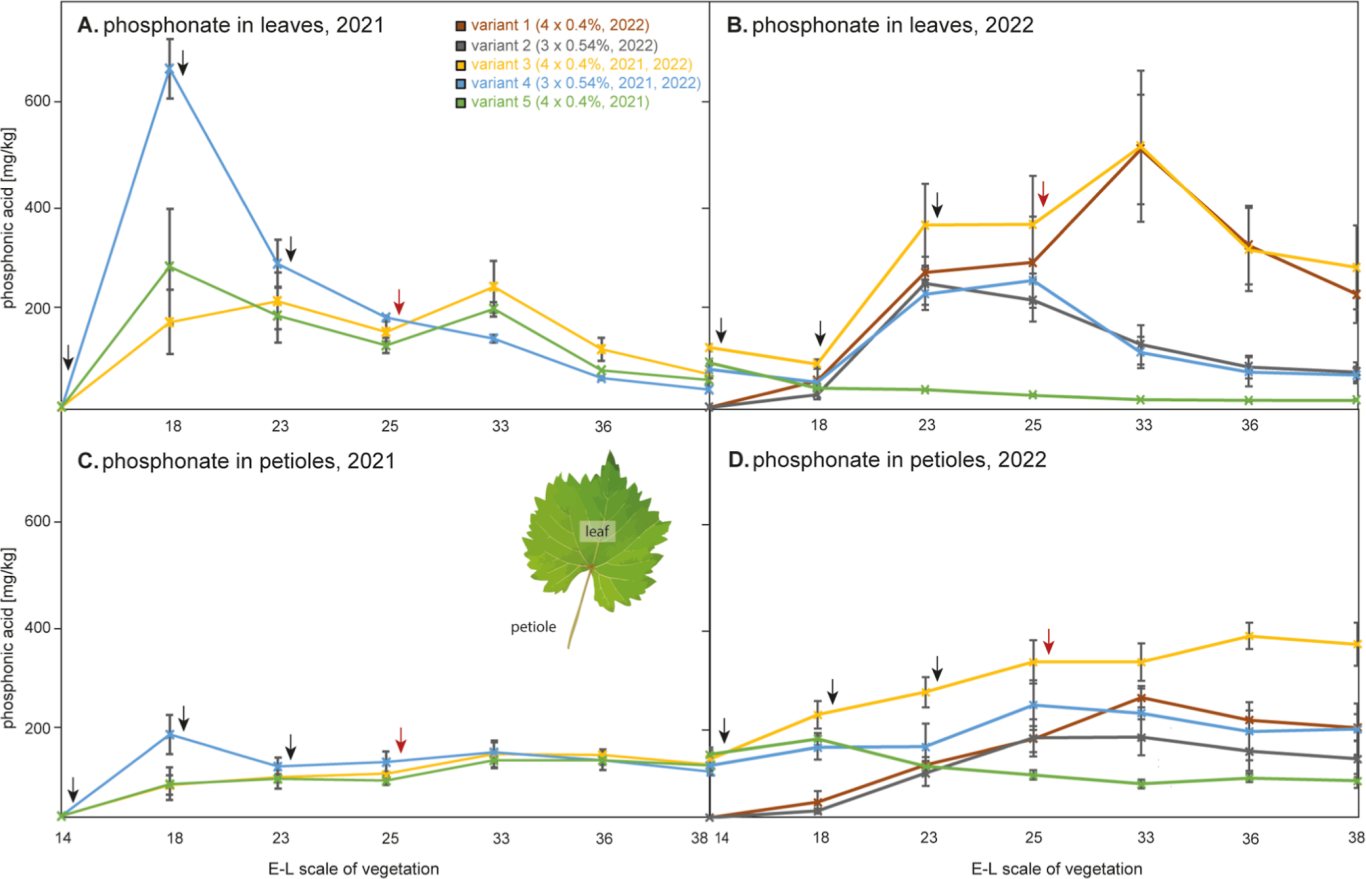
Phosphonate content (mg/kg, fresh weight) in leaves over the vegetation periods of (A) 2021 and (B) 2022 as well as in petioles at the same sampling times over the vegetation of (C) 2021 and (D) 2022. Application of phosphonate is as follows: green—four times 0.4% (only 2021); yellow—four times 0.4% (2021, 2022); blue—three times 0.54% (2021, 2022); red—four times 0.4% (2022 only); and gray—three times 0.54% (2022 only). A control with no application of the container vines was performed, and no residues were found (not shown). For the detailed description of the applications, see also Figure 1. The applications were performed shortly after the sampling in E-L 14, 18, 23, and 25 in each year. Arrows (black): applications of phosphonate for all variants, arrows (red): applications for variants 1, 3, and 5 only.

Figure 2 illustrates the variation in phosphonic acid content in the leaves and petioles of grapevines across different vegetation stages, as measured over two consecutive years, 2021 and 2022. After the first application of phosphonate in 2021 (Figure 2A), significant increases of phosphonate were observed in all variants. The application of a more concentrated (0.54% w/v) phosphonate solution in Variant 4 yielded a peak concentration of 652.8 ± 57.0 mg/kg leaves at stage 18, whereas applying a lower concentrated phosphonate solution (0.4% w/v) in Variants 3 and 5 only led to 163.8 ± 62.9 and 270.1 ± 110.9 mg/kg phosphonate. Following the peak after the first application, the phosphonate concentration steadily declined in Variant 4, where only 3 phosphonate applications were carried out. Spraying 4 times in Variants 3 and 5 led to a second peak concentration at a later vegetation stages (ca. E-L 33). The initially higher dosed variant reached slightly but significantly lower concentrations at the late vegetation stages expressed by a final concentration of 33.8 ± 3.7 mg/kg at stage E-L 38 for the higher dosed 3 times sprayed Variant 4, compared with 63.1 ± 20.0 and 51.8 ± 14.5 mg/kg for the lower dosed 4 times sprayed Variants 3 and 5, respectively. As indicated by the standard deviations, the biological variation of the average phosphonate levels was generally lower at the end of the vegetation seasons. For instance, those for Variant 5 decreased from 45.1 at stage E-L 23 to 4.7 mg/kg at E-L 25 and remained low thereafter in 2021.

Considering the area under the concentration vs time curve suggested that the overall uptake of phosphonate by the leaves of the plants of Variant 4 that had been sprayed 3 times concentratedly (AUC = 1182 ± 137 mg × wks/kg) was not significantly different from those of the 4 times sprayed plants of Variants 3 and 5 (1012 ± 169 and 946 ± 259 mg × wks/kg, respectively, Table 1).

**Table 1:** Area under the Concentration vs Time Curve (AUC) as the Approximation for Phosphonate Availability in Leaves and Petioles of Each Variant [mg × wks/kg]^b^

		1	2	3	4	5	6
		none in 2021	none in 2021	4 × 0.4%, 2021	3 × 0.54%, 2021	4 × 0.4%, 2021	3 × 0.54%^a^, 2021
variant, treatment 2021 and 2022		4 × 0.4%, 2022	3 × 0.54%, 2022	4 × 0.4%, 2022	3 × 0.54%, 2022	none in 2022	none in 2022
2021	leave	n.d.	n.d.	1012 ± 169^a^	1182 ± 137^a^	946 ± 259^a^	32 ± 6^b^
	petiole	n.d.	n.d.	769 ± 75^a^	850 ± 102^a^	691 ± 90^a^	119 ± 16^b^
2022	leave	1920 ± 438^a^	822 ± 200^b^	2210 ± 475^a^	868 ± 258^b^	181 ± 10^c^	
	petiole	1253 ± 58^a^	930 ± 217^a,c^	2439 ± 271^b^	1484 ± 400^a^	791 ± 32^c^	

a Application via soil. n.d., no phosphonate detected.

b Different letters indicate significant differences of means in each row (*p* < 0.05).

In the second year (Figure 2B), notable phosphonate residues were found in the leaves of all variants that had received phosphonate sprays in the previous year at the start of the vegetation season, i.e., at the first sampling point (E-L 14) and before initiation of treatments. Moreover, the concentration at E-L 14 in the second year was even higher in all variants than that found at the last sampling point of the previous year (e.g., 51.8 ± 14.5 in 2021 vs 87.6 ± 3.6 mg/kg in 2022, Variant 4). These findings suggest that the plant sourced and transferred significant amounts of phosphonate to the leaves either from residues in other perennial plant organs or from the soil that had been contaminated during spraying in the first year. After the first spraying treatment in 2022, phosphonate concentrations unexpectedly remained rather constant at 37.5–85.1 mg/kg in the leaves of Variants 3, 4, and 5, whereas those of Variants 1 and 2 receiving their first time sprays in 2022 increased from undetectable amounts to 25.7 ± 9.1 mg/kg and 52.7 ± 22.5 mg/kg at E-L 18, respectively. Subsequently, concentrations increased in all variants until E-L 25, but only the 4 times sprayed, lower dosed variants exhibited further increases until E-L 33. In contrast, phosphonate concentrations in the leaves of variants sprayed only 3 times but with a higher dose decreased already from E-L 25 onward. Ultimately, the phosphonate concentrations in leaves were substantially lower (63.3–69.4 mg/kg) at the final vegetation stage E-L 38 when the plants had been sprayed 3 times with a higher concentration than when sprayed 4 times with a lower concentration (220.0–272.4 mg/kg). The results do not indicate an accumulation of phosphonate residues over two years because the phosphonate concentrations in the leaves of Variant 3 (sprayed 4 times, two consecutive years) were not significantly different at the final vegetation stage from those of Variant 1 (sprayed 4 times, only 2022). Instead, the higher levels at the end of 2022 as compared to those at the end of 2021 might be the result of a high interyear variability. Specifically, a direct comparison of Variant 1 (sprayed 4 times, only 2022) with Variant 5 (sprayed 4 times, only 2021) allows unraveling the high interyear variability, which might have been caused by significantly lower precipitation in July and August (E-L 25–36) of 2022.^16^

In contrast to our observations in 2021, the overall phosphonate uptake by the leaves was more than twice as high in plants that had been sprayed 4-fold (Variants 1 and 3, AUC from 1920 to 2210 mg × wks/kg) as compared to that in the leaves of plants that had been sprayed 3 times with a more concentrated solution (Variants 2 and 4, AUC from 822 to 868 mg × wks/kg, Table 1).

Interestingly, phosphonate levels in the leaves of Variant 5, which was sprayed in 2021 but not in 2022, did only very slowly decrease from 87.6 to 14.4 mg/kg from E-L 14 to E-L 38, remaining widely unchanged between E-L 30 and E-L 38.

Figure 2C illustrates the phosphonate content in petioles during the 2021 growing season. By analogy to the data from leaves, phosphonate levels peaked in petioles at E-L 18 after the first application with the higher concentrated (0.54%) solution, reaching up to 180.8 ± 43.3 mg/kg in Variant 4 (blue line, Figure 2C). Nevertheless, this concentration was substantially lower than the corresponding concentration in the respective leaves (652.8 ± 57.0 mg/kg, Figure 2A). Spraying the plants of Variants 3 and 5 with less concentrated solutions (0.4%) led to only moderate increases in the petioles (68.3 ± 21.8 mg/kg) until E-L 18. In contrast to phosphonate levels in leaves, the levels in the petioles did not exhibit a subsequent decrease but remained widely unchanged until the end of the vegetation period.

The levels at the start of the subsequent vegetation period (Figure 2D) started at almost identical concentrations as they had ended in the previous year. Levels decreased when spraying in the second year was omitted (Figure 2D, green line) and increased when sprays had initiated (gray and red lines) or continued in the second year (yellow and blue lines). Here, the final concentrations at the end of the vegetation period were higher when plants had been sprayed 4 times with 0.4% phosphonate solutions (216.3 ± 32.6 mg/kg for Variant 1 and 418.3 ± 52.1 mg/kg for Variant 3) than when sprayed 3 times with 0.54% solutions (141.4 ± 43.9 mg/kg for Variant 2 and 213.0 ± 61.1 mg/kg for Variant 4). When treatments were discontinued in the second year (Variant 5, Figure 2D, green line), phosphonate levels declined slightly but remained at levels of 151.9–88.1 mg/kg higher than the levels found in the corresponding leaves (87.6–14.4 mg/kg, Figure 2B, green line).

The observations on overall phosphonate availability in the petioles were similar to those made for the leaves as described above. Overall, differences in AUCs were insignificant in 2021. In 2022, the AUCs were substantially higher for petioles from plants that had been sprayed 4 times as compared to those sprayed 3 times with a more concentrated solution (Table 1).

Data suggest that the concentration and number of phosphonate applications might allow modulating the levels accruing in both leaves and petioles. Considering a minimization of phosphonate residues in the plant itself, spraying more concentrated solutions at less time points might represent a promising way, if the plant strengthening effect of phosphonate was sufficient to maintain plant health even under increased fungal disease pressure. The latter remains to be investigated in further studies.

Our results align with those of other studies on horticultural crops. In greenhouse potatoes, foliar treatment resulted in approximately 500 mg/kg in leaves and 22 mg/kg in roots 48 h after application, indicating rapid downward translocation of phosphonate to the root tissue.^18^ Borza et al.^19^ examined the effects of foliar applications using lower (0.23% solution of mono- and dipotassium salts of phosphonic acid; 10 mL/plant) versus higher amounts (0.46%, 10 mL/plant) with two and four applications for each concentration. Similar to our methodology, foliar spraying was performed weekly, with sampling 1 week after the final application. When comparing the results of the 4-fold application of the 0.23% against those of the 2-fold application of the 0.46% solution, they found 492.0 and 444.2 mg/kg in leaves, respectively, compared to our findings that spraying higher concentrated solutions at less time points led to lower residues in the leaves.

Another study investigated phosphonate levels in the leaves and stems of *Eucalyptus* plants after a single spraying application of 0.5% and 1.0% phosphonate solutions. The phosphonate concentrations in leaves and stems were 854 and 1760 mg/kg when spraying the 0.5% solution and 1863 and 2010 mg/kg for the 1.0% solution, respectively.^20^

Guo et al.^21^ conducted greenhouse trials with soybeans using a 0.45% (w/v) phosphonate solution, resulting in significant phosphonate accumulation in the leaves (35.3 g/kg) and stems (12.6 g/kg) 48 h after treatment, supporting our findings on the distribution to leaves and stems in grapevine after foliar application. One week after foliar application, the levels of phosphonate in avocado leaves and stems were 42 and 21 mg/kg, respectively. After 8 weeks, these levels shifted to 19 mg/kg in leaves and 75 mg/kg in stems, demonstrating the high mobility of phosphonate.^22^ Similarly, our study suggested a basipetal migration from leaves, comparing E-L 23 to 33 of leaves and petioles in Variant 2 in 2022 (cf. Figure 2B,D).

In a study on coconut trees by Yu et al.,^23^ phosphonate concentrations were initially observed to increase in the petiole and rachis following trunk injection, spreading throughout the leaf. After 40 weeks, the concentrations were higher in the spear leaf (280 mg/L) than in the petiole. A similar but less pronounced distribution pattern and presumed xylem mobility, showing acropetal migration, was observed in our soil-treated Variant 6 (see below). In spite of these similarities, results from other crops should be considered with caution.

### 3.2 Phosphonate Uptake from Soil

**Figure 3:**
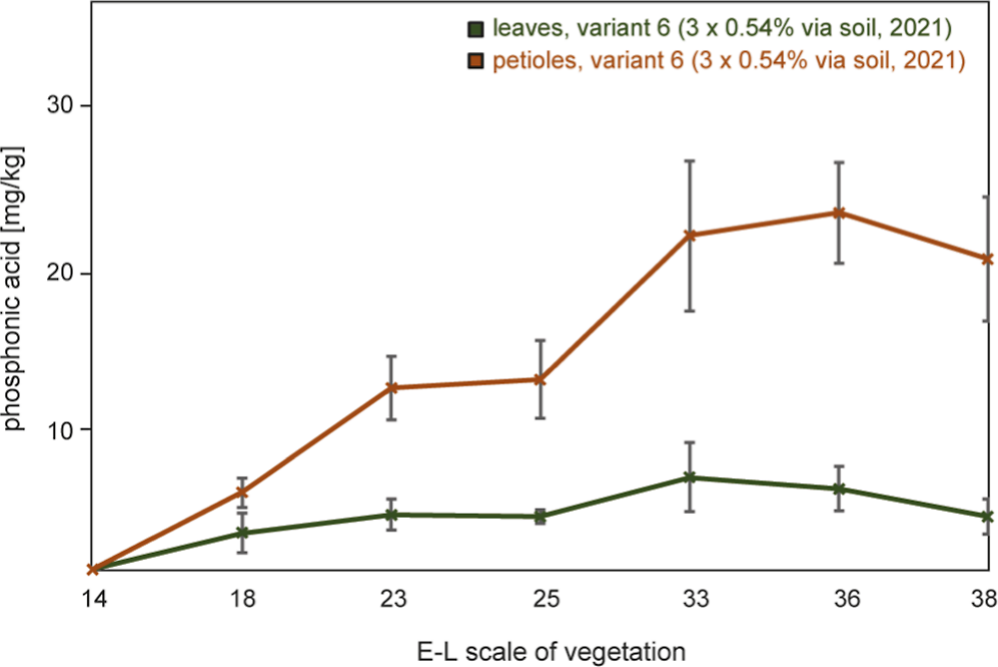
Phosphonate content (mg/kg, fresh weight) in leaves and petioles in 2021 of treatment Variant 6 (3 × 0.54%, 2021, applied to the soil only).

A relatively low uptake and distribution of phosphonate from the soil-treated variant (6) was observed, as shown in Figure 3. Interestingly, the phosphonate content in the leaves and petioles of this variant showed an inverted ratio compared to foliar application. Specifically, the final phosphonate content at E-L 38 was 3.5 ± 1.2 mg/kg in leaves and 20.7 ± 4.1 mg/kg in petioles. This pattern differed from that of foliar application where the levels in the leaves were higher than those in the petioles. The phosphonate concentration in petioles consistently exceeded the one in leaves over the entire vegetation period with the difference becoming more pronounced with fruit maturity. In accordance, the areas under the concentration vs time curve are ca. 3.6-fold higher for the petioles (ca. 119 mg × wks/kg) than for the corresponding leaves (32 mg × wks/kg, Table 1 and Figure 3). As a consequence of soil phosphonate, well detectable concentrations (>5 mg/kg) can still be transferred to the berries until harvest (see below), potentially leading to regulatory implications in organic viticulture. Based on these observations, we had earlier proposed an index to allow tracing the origin of inorganic phosphonate from either foliar application or another source such as soil or vine residues. Detailed information about this index and further findings can be found in our previous report.^13^

### 3.3 Effect of Phosphonate Treatment on Residues in Berries

**Figure 4:**
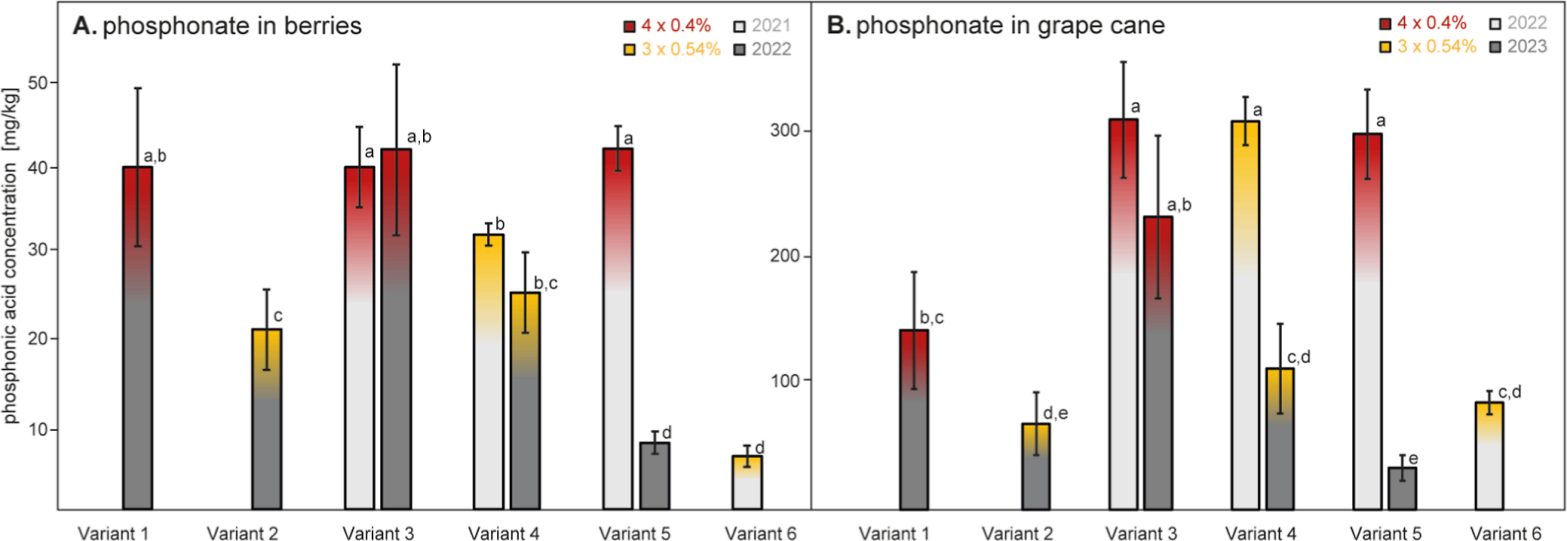
(A) Phosphonate content (mg/kg, fresh weight) in berries harvested at the phenological stage of E-L 38 in 2021 (light gray) and 2022 (dark gray) of each discussed treatment variant (cf. Figure 1), differing in the number of applications (4 or 3 times) and the concentration of phosphonic acid in the spray solution (0.4 or 0.54%, respectively). (B) Phosphonate content (mg/kg, fresh weight) in grape canes, sampled in February 2022 (light gray) and 2023 (dark gray). Please note that Variant 5 was not treated in 2022. Variant 6 had received phosphonic acid solely from the soil (3 × 0.54%) and only in 2021. Different letters indicate significant differences of means at *p* < 0.05.

Considering the relevance of residues in the final product, berries of all treatment variants were harvested after E-L 38 and analyzed as described above (Figure 4A). Results regarding the control Variant 7, whose plants had not received phosphonate applications, are not shown because no residues were found in both years. Variants 1, 3, and 5 sprayed 4 times with 0.4% phosphonate solutions yielded berries with significantly higher concentrations (ca. 39–41 mg/kg, Figure 4) than berries of Variants 2 and 4 sprayed only 3 times with 0.54% phosphonate solutions (ca. 20–31 mg/kg). These findings are in line with the above-mentioned observations for leaves and petioles. That is, plants of the variants sprayed 3 times with higher concentrations (0.54%) exhibited lower phosphonate residues at the end of the vegetation periods than those sprayed 4 times with lower concentrations (0.4%).

In those variants treated in two consecutive years (Variants 3 and 4), a cumulative effect was not observed, as shown by insignificant differences in phosphonate levels (Figure 4). However, a depletion effect was observed for the berries of Variant 5 which contained substantially higher phosphonate levels (40.6 ± 2.5 mg/kg) when harvested in the year of active application (2021) than when harvested in the subsequent year when plants had not been sprayed (7.5 ± 1.3 mg/kg).

Additionally, Variant 6 demonstrated that a soil contaminated with phosphonates led to berries with considerable phosphonate concentrations of approximately 6.0 ± 1.2 mg/kg which unambiguously demonstrates phosphonate uptake from the soil to the berry (Figure 4). This finding is particularly important for ecological farming, where the use of phosphonates is not permitted due to the lack of approval in organic agriculture.

The intake of phosphonate in avocado fruits was studied by McLeod et al.^24^ and Masikane et al.^25^ where the latter recorded an intake to a final concentration of 9.3 mg/kg in fruits following two foliar applications with 2% potassium phosphonate in fall and summer. McLeod et al.^24^ conducted three foliar applications with 0.6% potassium phosphonate in the fall and two applications with 0.5% in the summer on avocado trees, including two variants where spraying was carried out with the total volume or 3/4 of the total volume. Spraying the full volume yielded about 55 mg/kg, while the reduced 3/4 volume yielded 65 mg/kg of phosphonate residues in fruits.

Malusà and Tosi^26^ investigated phosphonate accumulation and depletion in apple trees over three consecutive years (2000–2002). They reported phosphonic acid levels of 1.55, 6.36, and 5.86 mg/kg apple fruit in one trial field and 1.70, 7.62, and 8.49 mg/kg in another trial field after applying the commercial fungicide Aliette, containing 800 g/kg Fosetyl-Al. Following the suspension of the treatments, they observed a decline in phosphonic acid levels over a period of three years from 1.55 to 0.18 and finally to 0.10 mg/kg, representing a decrease of 88% and 45% each subsequent year. In the current study, a decrease in phosphonate content from 40.6 to 7.5 mg/kg observed (Variant 5) 1 year after treatment discontinuation, equaling a decrease by ca. 82% (see above).

Generally, berry residues reported in this work (Figure 4A) correspond with the results of a large observational study on commercial grape samples, which had reported 1.0 mg/kg (with a maximum of 120 mg/kg) in perennial fruits and 4.3 mg/kg (with a maximum of 50.8 mg/kg) in wine samples from integrated production.^11^

### 3.4 Phosphonate Residues in Other Vine Compartments

Phosphonate residues in grape canes (Figure 4B) sampled in February of both 2022 and 2023 only partly correlated with the residues found in the berries harvested in the preceding years 2021 and 2022 (cf. Figure 4A and B, coefficient of determination *R*_linear_^2^ = 0.63). Rather, phosphonate levels in the canes depended on the treatment and the year of application as described above for the berries. In canes sampled in 2023, phosphonate levels of Variants 1 and 3 treated 4 times with 0.4% phosphonic acid were higher (142.6 ± 46.5 mg/kg and 232.8 ± 64.7 mg/kg) as compared with those of Variants 2 and 4 treated 3 times with 0.54% phosphonic acid (68.5 ± 24.9 mg/kg and 112.2 ± 35.6 mg/kg), similar to the analogous variants of berry residues from 2022.

Notably, the phosphonate residue levels were found to be 4 to 6 times higher in grape canes than those in the corresponding berries. For instance, treatment Variants 3, 4, and 5 showed the highest phosphonate concentrations in 2022 where concentrations exceeded 300 mg/kg.

These observations in grape canes are consistent with results reported by Yu et al.^23^ who applied a substantially higher dosage of phosphonate (e.g., 25.2 g/180 mL equivalent to 14% potassium phosphonate) to coconut trees, observing a greater accumulation and persistence of phosphonate in certain tissues over time. They observed that phosphonate concentrations in palm tissues were sustained at high levels, particularly in basal rachis tissues (above 200 mg/L), for up to 60 weeks after trunk injection.^23^

**Figure 5:**
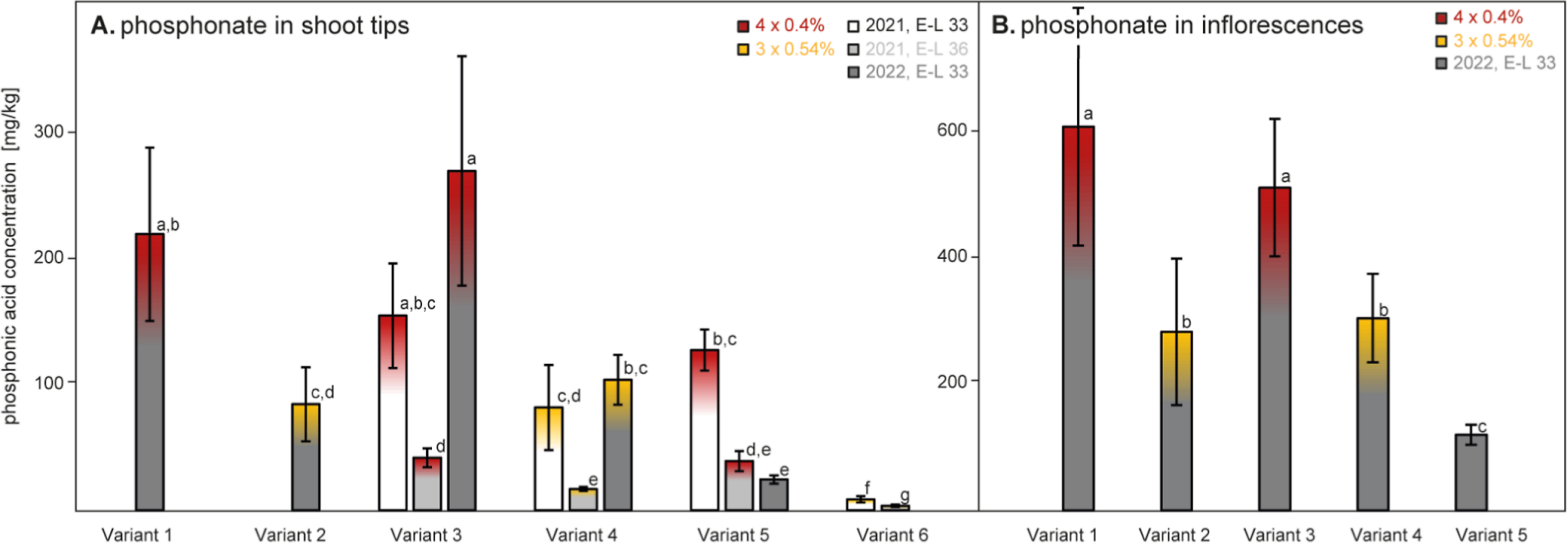
(A) Phosphonate content (mg/kg, fresh weight) in shoot tips harvested at the phenological stage of 33 and 36 in 2021 (white and light gray, respectively) and stage of 33 in 2022 (dark gray) of each discussed treatment variant (cf. Figure 1) differing in the number of applications (4 or 3 times) and the concentration of phosphonic acid in the spray solution (0.4 or 0.54%, respectively). (B) Phosphonate content (mg/kg, fresh weight) in inflorescences, sampled at E-L 23 in 2022. Please note that Variant 5 was not treated in 2022. Variant 6 had received phosphonic acid solely from the soil (3 × 0.54%, 2021). Different letters indicate significant differences of means at *p* < 0.05.

As shown in Figure 5, phosphonate residues in other compartments, such as shoot tips (Figure 5A) and the inflorescence (Figure 5B), widely followed the pattern described above for leaves and petioles. Spraying 3 times a concentrated (0.54%) solution in Variants 2 and 4 yielded consistently lower phosphonate residues as compared to spraying 4 times a less concentrated (0.4%) solution (Figure 5).

In brief summary, our study provides detailed insights into the spatial distribution and concentrations of inorganic phosphonate in different grapevine compartments following different application methods, i.e., spraying 4 times with 0.40% versus 3 times with 0.54% phosphonic acid during two consecutive years, as well as from phosphonate uptake from the soil. Foliar application significantly increased phosphonate concentration in leaves and petioles, with a notable carryover effect across seasons. Higher concentration (0.54%) with 3 applications generally resulted in lower residues compared to using lower concentrations (0.4%) applied 4 times in all studied compartments (leaves, petioles, canes, berries, shoot tips, and inflorescence). Although soil applications led to a comparably low overall uptake, they still contributed to measurable residues in berries. Notably, phosphonate concentrations in petioles were higher than in leaves in cases of soil uptake, indicating acropetal translocation of phosphonate. The persistence of phosphonate in grape canes supports the notion that phosphonate can be effectively retained in plant tissues for an extended period.

A limitation of our study was the use of pure phosphonic acid instead of commercial, authorized potassium phosphonate formulations. Future studies beyond studying the distribution of inorganic phosphonate, e.g., investigating the antifungal efficacy of the applications, should use commercially available and authorized products.

Further research should now evaluate the efficacy of the aforementioned phosphonate treatments to strengthen the plant and improve its defense against fungal attacks. Possibly, further fine-tuning of the balance between effective disease management and minimal residue levels is warranted. Long-term field studies should also be conducted to confirm or disprove our findings on container-grown vines under highly variable, open-field conditions, which could further enhance sustainable viticulture practices.
